# Supplementary figures and images for: Dissecting new genetic components of salinity tolerance in two-row spring barley at the vegetative and reproductive stages
Source: PLoS One. 2020 Jul 23;15(7):e0236037. doi: 10.1371/journal.pone.0236037 (PMC7377408; doi:10.1371/journal.pone.0236037)

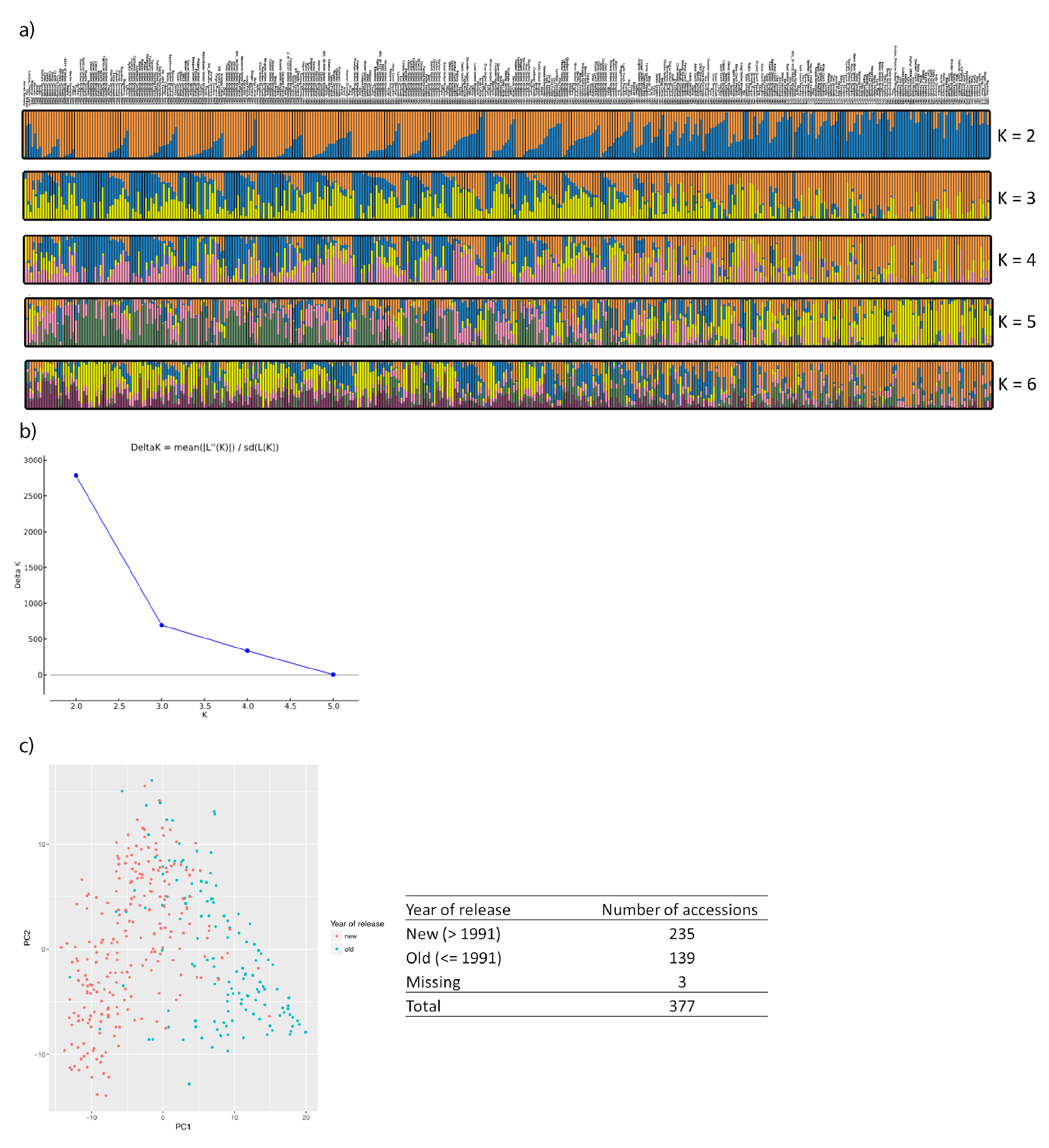

Supplement: S1 Fig — a) We ran STRUCTURE for K from 1 to 6. Each vertical bar represents an accession. The labels from closest to furthest from the plot are year of release, country of origin, and name of the 377 accessions. b) K = 2 was chosen as the best K based on the ΔK method [33] using HARVESTER STRUCTURE software. c) PC1 and PC2 generated by GAPIT are plotted and colored by the year of release of the accession. The year 1991 was used as the cutoff to separate “old” from “new” accessions in this graph. At the bottom, the result from the STRUCTURE software for K = 2 is shown, ordered by year of the accession’s release. (TIF) [file pone.0236037.s001.tif]

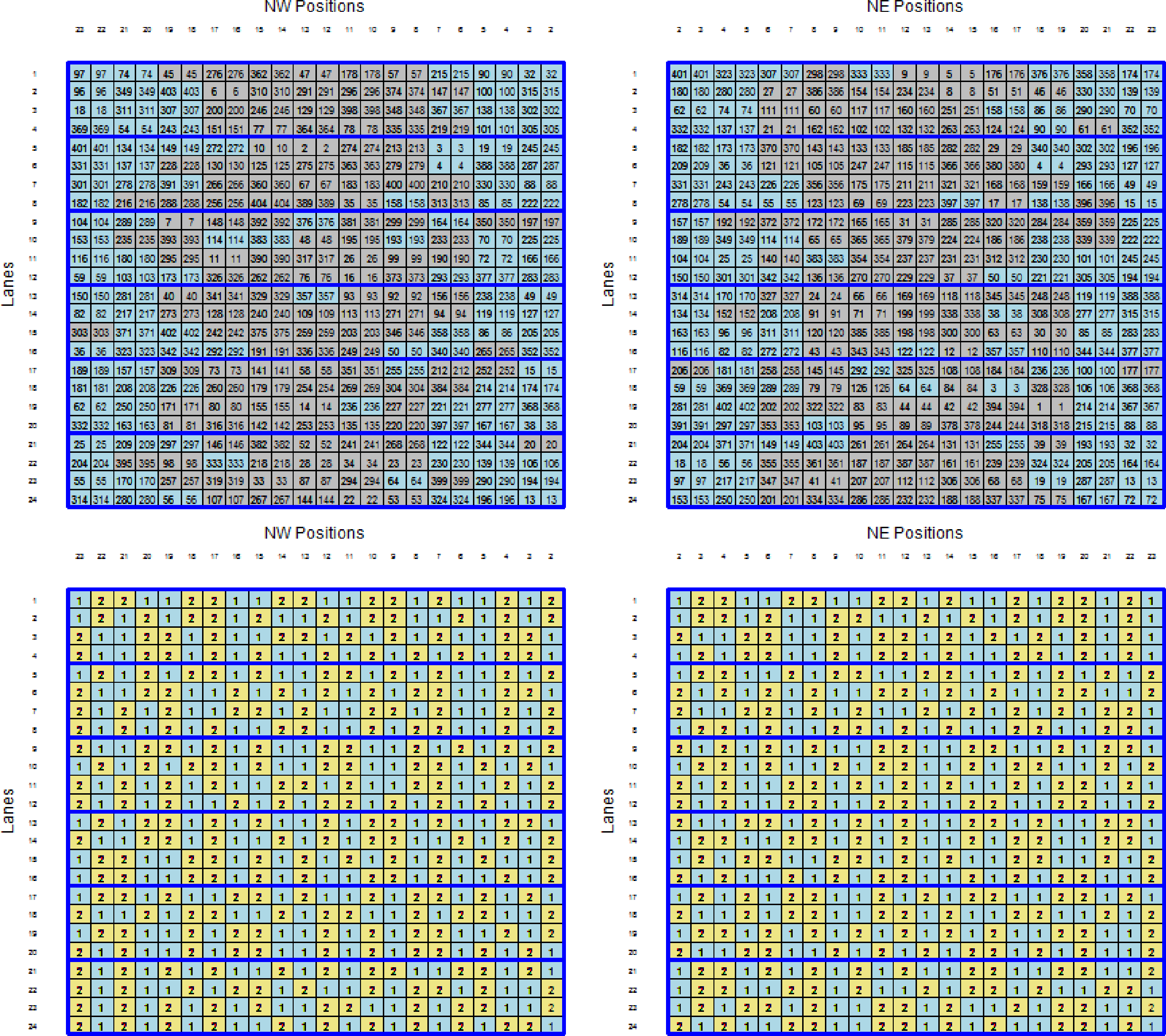

Supplement: S2 Fig — In top panels, allocation of replicated (blue), unreplicated (grey) accessions. In bottom panels, allocation of control (blue, 1) and salt (yellow, 2) conditions. The design is a split-plot design where each pair of carts corresponds to a main plot to which an accession is allocated. The left and right panels correspond to the design in the Northwest (NW) and Northeast (NE) smarthouses, respectively. Each position in a smarthouse corresponds to an individual plant. (TIF) [file pone.0236037.s002.tif]

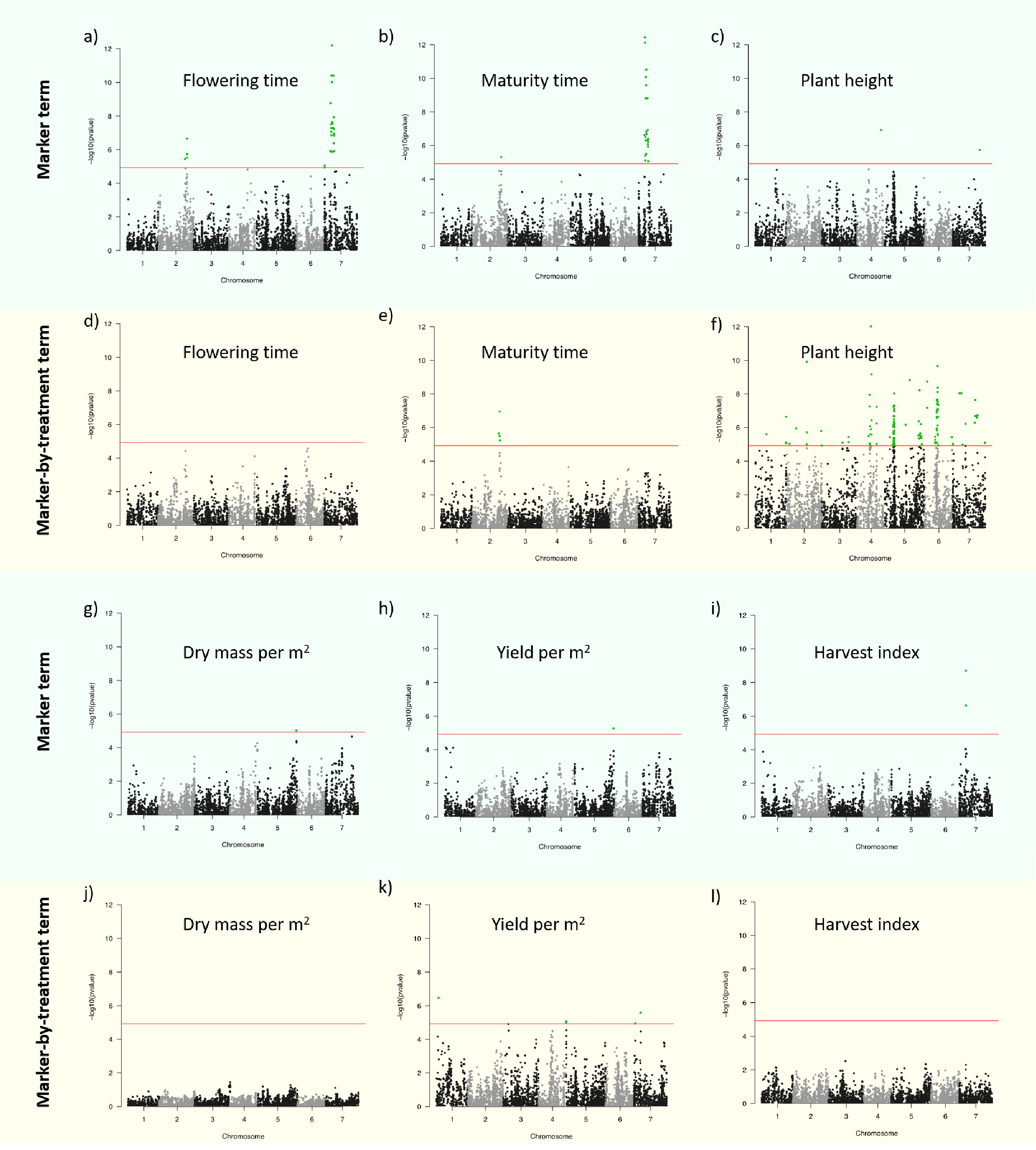

Supplement: S3 Fig — Results for flowering time (a, d), maturity time (b, e), plant height (c, f), dry mass per m2 (g, j), yield per m2 (h, k), and harvest index (i, l). Green- and yellow-shaded panels illustrate the marker and marker-by-treatment models, respectively. Loci significant in the marker term contribute to the traits regardless of the treatment, whereas loci significant in the marker-by-treatment term are responsive to salinity treatment. The red line indicates the Bonferroni corrected p-value threshold above which SNPs are significant (indicated by green dots). (TIF) [file pone.0236037.s003.tif]

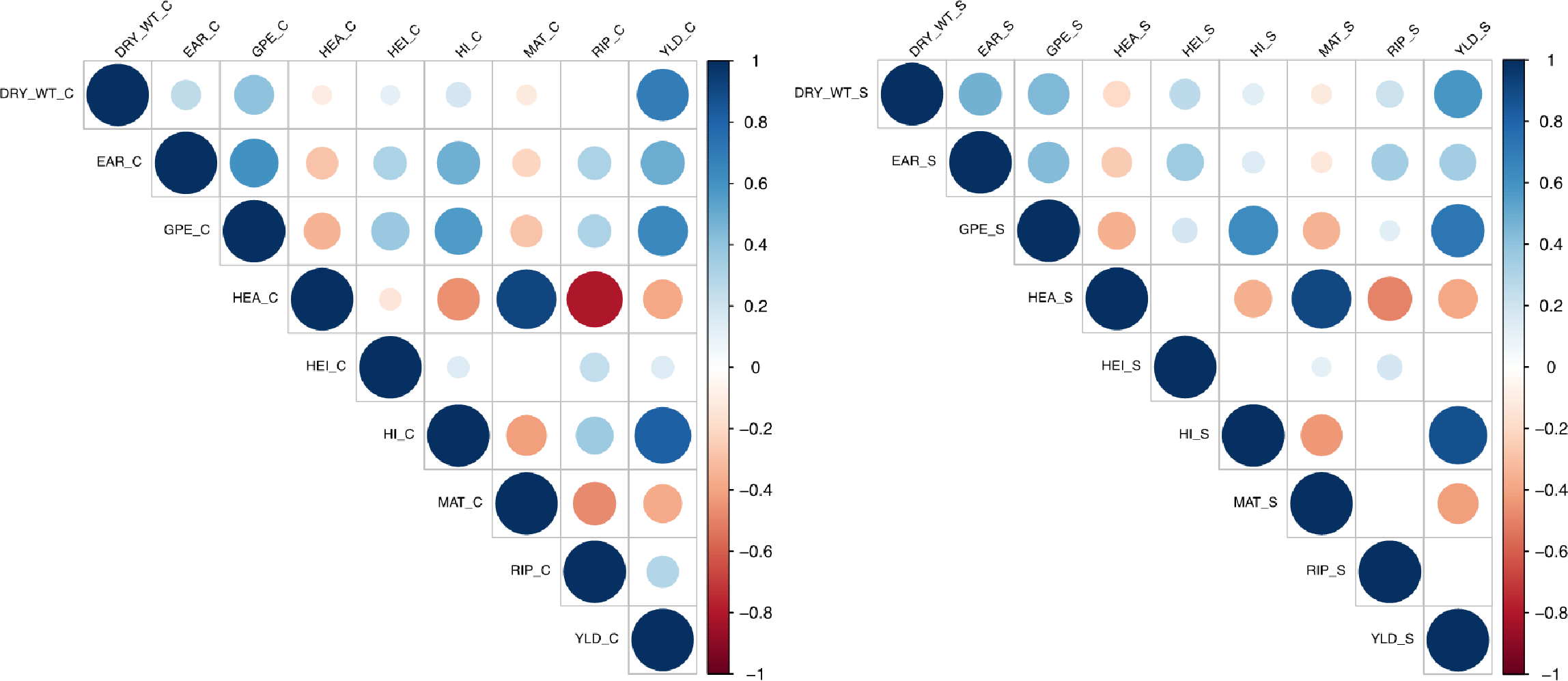

Supplement: S4 Fig — The size and the color of the circles refer to the strength and the significance of the correlation, respectively. Non-significant correlations are indicated by blank cells. (TIF) [file pone.0236037.s004.tif]

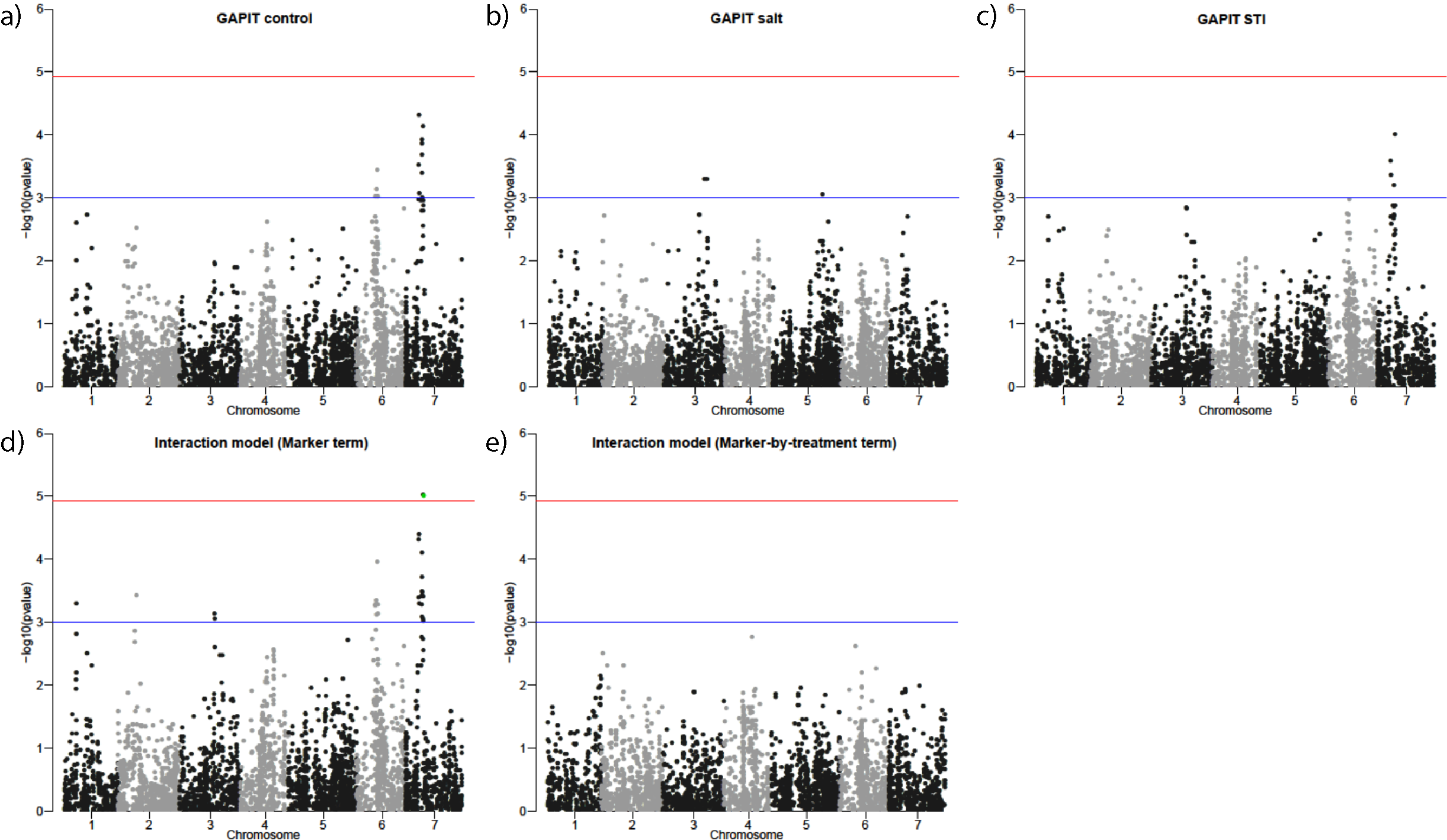

Supplement: S5 Fig — Manhattan plots of RGR 1to4 for a) control using GAPIT b) salt using GAPIT c) stress tolerance index STI (as defined in Fernandez [56]) using GAPIT d) marker term of interaction model e) marker-by-treatment term of interaction model. Loci significant in the marker term contribute to the traits regardless of the treatment, whereas loci significant in the marker-by-treatment term are responsive to salinity treatment. (TIF) [file pone.0236037.s005.tif]
